# Non-Destructive Technologies for Detecting Insect Infestation in Fruits and Vegetables under Postharvest Conditions: A Critical Review

**DOI:** 10.3390/foods9070927

**Published:** 2020-07-14

**Authors:** Akinbode A. Adedeji, Nader Ekramirad, Ahmed Rady, Ali Hamidisepehr, Kevin D. Donohue, Raul T. Villanueva, Chadwick A. Parrish, Mengxing Li

**Affiliations:** 1 Department of Biosystems and Agricultural Engineering, University of Kentucky, Lexington, KY 40546, USA; Nader.Ekramirad@uky.edu (N.E.); radyahme2@gmail.com (A.R.); aha322@g.uky.edu (A.H.); mengxingli0324@gmail.com (M.L.); 2 Department of Biosystems and Agricultural Engineering, Alexandria University, Alexandria 21526, Egypt; 3 Department of Electrical and Computer Engineering, University of Kentucky, Lexington, KY 40506, USA; kevin.donohue1@uky.edu (K.D.D.); chad.parrish@uky.edu (C.A.P.); 4 Department of Entomology, University of Kentucky, Princeton, KY 42445-0469, USA; raul.villanueva@uky.edu

**Keywords:** non-destructive detection, noninvasive technology, insect infestation, post-harvest technology, online monitoring, fruits, vegetables

## Abstract

In the last two decades, food scientists have attempted to develop new technologies that can improve the detection of insect infestation in fruits and vegetables under postharvest conditions using a multitude of non-destructive technologies. While consumers’ expectations for higher nutritive and sensorial value of fresh produce has increased over time, they have also become more critical on using insecticides or synthetic chemicals to preserve food quality from insects’ attacks or enhance the quality attributes of minimally processed fresh produce. In addition, the increasingly stringent quarantine measures by regulatory agencies for commercial import–export of fresh produce needs more reliable technologies for quickly detecting insect infestation in fruits and vegetables before their commercialization. For these reasons, the food industry investigates alternative and non-destructive means to improve food quality. Several studies have been conducted on the development of rapid, accurate, and reliable insect infestation monitoring systems to replace invasive and subjective methods that are often inefficient. There are still major limitations to the effective in-field, as well as postharvest on-line, monitoring applications. This review presents a general overview of current non-destructive techniques for the detection of insect damage in fruits and vegetables and discusses basic principles and applications. The paper also elaborates on the specific post-harvest fruit infestation detection methods, which include principles, protocols, specific application examples, merits, and limitations. The methods reviewed include those based on spectroscopy, imaging, acoustic sensing, and chemical interactions, with greater emphasis on the noninvasive methods. This review also discusses the current research gaps as well as the future research directions for non-destructive methods’ application in the detection and classification of insect infestation in fruits and vegetables.

## 1. Introduction

In recent years, there has been significant growth in the consumption of fruits and vegetables, which can be attributed to several factors, among which is increased awareness of their health benefits [1]. Consumers, especially the “Generation Z” (post-millennial with ages between 11 and 23 years) that constitute about 32% of the US population, are more cognizant of what they eat and many of them tend to eat healthy, often preferring organic foods [2]. The easy access to information through smart devices has also increased the understanding of consumers on what they eat, and many more people, beyond the younger generations, are tending toward more natural, minimally processed, and organic food. This demand is driving the trend for high-quality, consistent, and safe products at a reasonable price [3]. The agricultural production sector and the food industry as well as the safety agencies are saddled with the responsibility to meet these increasing demands for produce with low-toxicity pesticides. In order to be efficient in meeting quality and demand, there is a need to replace destructive and off-line conventional quality assessment methods with rapid, non-invasive, environmentally friendly, and accurate methods for quality assessment and safety assurance [4].

Insects cause enormous damage to fruits and vegetables each year, leading to major production and economic losses in the agricultural production and food industry worldwide. Insect pests are considered to be responsible for approximately 10–20% of yield losses in major crops worldwide, and even far more in developing countries, reaching about 50% of annual horticultural production in Africa, which is a $22.5 billion industry [5]. The havoc caused by insect pests in trans-border trade, with increased global trade network, is enormous. The detection of these insect pests before they get into the supply chain is still a major challenge for the industry. The US loses about $40 billion yearly because of these organisms of quarantine concern [6,7]. On the other hand, insect pests such as budworms are hard to control [8]. Insect feeding often cryptically occurs within fruits and vegetables without showing an obvious external symptom until they are nearly fully rotten. This is the case of the codling moth (*Cydia pomonella*, Lepidoptera: Tortricidae), one of the most devastating pests in apples. This insect has four main stages in its life cycle, egg, larva, pupa, and adult moth [9]. The larval phase is its most devastating phase when it feeds on the flesh and pulp of fruits it was laid on. When the point of entry is the calyx, the damage is difficult to detect with the subjective method of assessment common in most apple processing plants and this is why non-destructive detection becomes important [9,10]. Early detection when eggs are laid on the surface of the produce is also very important.

In order to prevent the economic and ecological losses from alien insect pests, increasingly stringent quarantine measures are being put in place by governments. As an example, Fruits and Vegetables Import Requirements (FAVIR) of the US government require preclearance of horticultural consignments in the exporting countries as well as inspections at the ports of arrival for any live larva or pupa of quarantine pests. In general, a biometrically designed statistical sampling is applied to conduct phytosanitary physical inspections against any quarantine-significant insect in fruits and vegetable commodities. In 2017, around 194 million pounds of fresh fruits and vegetables were inspected and cleared for shipment to the United States [11]. Based on the United States Department of Agriculture (USDA) report about the US plant inspection stations in 2017, the inspection of plant materials is mostly conducted physically, along with some modern technologies such as digital imaging, X-ray and molecular detection tools for low-volume plants, plant cuttings, and seeds. As a result, automatic, fast, and reliable noninvasive methods of detection are needed to monitor quarantine pest existence and the internal quality of the fruits and vegetables in high-volume shipments [12].

The rapid advancement in electronic technology and data analytics with greater computing power, along with their increased application in the agricultural field, have introduced new methods for non-destructive quality assessment of fruits and vegetables. A range of techniques have been reported for non-destructive detection of insect infestation such as near-infrared (NIR) spectroscopy [13,14,15,16], acoustic methods—sound/noise/vibration [17,18,19], imaging—visible light sensing [20], hyperspectral imaging [3,21], nuclear magnetic resonance [22], X-ray [23,24], volatile emission, and others [25,26,27,28]. With these new applications of technology in agricultural processing as well as the multiplicity of investigations all over the world, up-to-date reviews are needed as an orientation over technological applications in agriculture and food science. There are currently few reference papers reviewing some of the state-of-the-art works in non-destructive quality assessment of fruits and vegetables. Particularly, no review is available focusing on recent postharvest non-destructive methods for the detection of insect infestation in fruits and vegetables. Thus, this paper reviews all known techniques used for postharvest non-destructive detection of internal insect infestation in fruits and vegetables: their basic principles of operation are explained, the merits, as well as the limitations of each method are profiled, several examples of applications are presented, and challenges and opportunities for the future are discussed.

## 2. Traditional Manual Methods

Traditionally, the insect identification methods in horticultural products are mostly manual and destructive in nature and based on external morphology, like defects, color and size, protein analysis like the enzyme electrophoretic discrimination, and molecular tools such as deoxyribonucleic acid (DNA) analysis [11,29]. Thus, most of the traditional methods applied for quality assessment of fruits and vegetables are time-consuming, labor-intensive, tedious, cost-intensive, and subjective [30]. On the other hand, manual and destructive methods of quality evaluation are not suitable in industries such as the packaging industry as it ruptures the fruit tissue and evaluation of a complete sample cannot be done. For example, the detection of internal defects is carried out directly by manual destructive sampling and searching for defects or indirectly by correlating the results obtained on assessing other chemical or physical characteristics, for instance, measurement of ripening is based on color or firmness.

Non-destructive methods are more effective than traditional conventional methods because they are based on physical properties that correlate well with certain quality factors of fruits and vegetables [30]. In addition, non-destructive methods are advantageous over traditional destructive methods as they do not rupture the fruit tissue and can be used to assess internal structures and quality of fruits and vegetables. It is useful in sorting superior quality fruits and vegetables from substandard ones based on their size and shape in the online system and sampling of all fruits or vegetables is carried out, which ensures maximum quality.

## 3. Noninvasive Methods

### 3.1. Spectroscopic Techniques

Spectroscopy methods provide operational information about the chemical and physical characteristics of fruits and vegetables by obtaining reflectance, transmittance, absorbance, or scattering of polychromatic or monochromatic radiation from the surface of the sample in the ultraviolet (UV), visible (Vis), and NIR regions of the electromagnetic spectrum. But, the application of NIR region (780 to 2500 nm) is particularly compelling because it is sensitive to overtones and combinations of chemical bonds such as C–H, O–H, and N–H, which are abundantly present in foods. Moreover, NIR spectroscopy has the capacity of measuring multiple quality attributes of foods simultaneously [28]. Some researchers have proven the high potential of NIR spectroscopy for the detection of insects or insect damage in food commodities, such as blueberries [14], cherries [31], figs [32], green soybeans [33], jujubes [16], chestnuts [34], and other foods [25,26,27,35].

While the technical configurations of the equipment used for spectroscopy, such as sensor type and resolution, affect the measurement, the two most significant factors affecting the detection of insect infestation are wavelength range and optical measurement mode (interactance, reflectance, and transmittance) [36]. According to a recent meta-analysis conducted by Jamshidi [36], summarizing different studies for non-destructive detection of internal insect infestation in fruits using the spectroscopy technique, the spectral range of visible/shortwave near-infrared (350–1100 nm) showed lower classification accuracy compared to NIR or Vis/NIR (total error of 21.71% in comparison to errors of 13.30%, or 13.65%, respectively). Furthermore, the results showed that applying the interactive mode for spectroscopy achieved lower errors in classifying infested fruits from healthy ones (error of 6.66% compared to errors of 15.73% and 16.04% for reflectance and transmittance modes, respectively) [36].

In fact, the detection of insect infestation by NIR spectroscopy can be achieved through either direct detection of insects and larvae due to their hemolymph, lipids, and chitin content [34], or indirect identification of the changes in the spectral properties of infested tissues resulting from internal browning or darkening, dehydration, or microbial contamination. Since NIR spectra (especially at the short wavelength and high-frequency region of 850 to 1888 nm) are capable of penetrating the fruit peel and tissue, useful information can be acquired by measuring the interaction (energy attenuation) between the IR energy and the food samples. On the other hand, the high moisture content of fruits and vegetables makes it difficult for the light in the long wavelength near infrared range of 1100–2500 nm to penetrate through the whole fruit, especially in very large samples. Consequently, the short wavelength NIR spectroscopy is normally used in the internal quality assessment of fruits to detect the presence of insects via changes of chemical and optical properties of whole fruit caused by insect infestation.

As an example, Xing et al. [31] applied visible and NIR spectroscopy (550–980 nm), in the transmittance mode, to detect plum curculio (*Conotrachelus nenuphar*) infestation in tart cherries and achieved an overall detection accuracy of 82–87%. Their spectral analysis showed that the maturity level of tart cherry has some effects on the classification accuracy because the over-ripened cherries have similar spectral characteristics as the infested tissues. As a result, they suggested that the classification accuracy for the samples harvested at the right time is better than that for the late-harvested samples. Additionally, they declared that total soluble solid (TSS) and firmness could be complementary factors for explaining the difference between the insect-infested and intact tart cherries in order to build better classification models. It shows that investigating the correlation between internal quality attributes relating to insect infestation, such as firmness and TSS, as well as calculating and using spectral indices combining the spectra at two or more wavelengths, can improve the classification accuracy in spectroscopic methods. In another work, Peshlov et al. [14] examined three NIR instruments in diffuse reflectance mode in spectral ranges between 600 and 1700 nm, for classifying fruit fly larvae damages in wild blueberries. The three instruments showed different infestation detection accuracies between 58% and 82%. Wang et al. [16] used three NIR sensing modes (i.e., reflectance, absorbance, and transmittance) in different spectral regions of 400 to 2000 nm for the detection of insect infestation in jujubes. They reported that absorbance for 1000–2000 nm and transmittance for 400–1000 nm gave better results than reflectance mode. Saranwong et al. [15] evaluated the possibility of the use of NIR spectroscopy for non-destructive detection of fruit fly eggs and larvae in intact mangoes at different infestation levels. The best classification was attained applying spectra of green mangoes obtained after 48 h of infestation, and this has an error rate of 4.2% for infested fruit and 0% for the control fruit. In another study, Biancolillo et al. [34] demonstrated the feasibility of using NIR spectroscopy to detect hidden damages by Indian-meal moth (*Plodia interpunctella*) in stored rice sourced from six different countries in Asia and Europe. They applied partial least square discriminant analysis (PLS-DA) and soft independent modeling of class analogies (SIMCA) analysis classification methods and achieved the classification rate between 95.6% of the edible 97.5%, with SIMCA proving to be more sensitive. In a recent study, Jamshidi et al. [37] investigated the possibility of using Vis/NIR spectroscopy combined with pattern recognition methods (PCA-DA) to detect pomegranate fruits with internal infestation caused by Carob Moth (*Ectomyelois ceratoniae*) larvae. Their results showed a total classification rate of 90.6% for test data, suggesting that Vis/NIR spectral data collected from the calyx region of pomegranate fruit and analyzed by the PCA-DA method can provide useful information for non-destructive detection of internally damaged pomegranates by Carob Moth.

### 3.2. Visible Light Sensing

In the last four decades, machine vision systems have been extensively investigated to replace the human role in several agricultural applications, including sorting, detecting defects and diseases, and characterizing other quality attributes of agricultural products [38,39]. Visible light sensors at a wavelength from 380 to 750 nm falls in the range that is generally used for detecting external or surface features [8].

A glossary of studies where computer vision was used for detecting insect infestation in fruits and vegetables are shown in Table 1. Blasco et al. [40,41,42] implemented several studies for assessing the Thrips (Thysanoptera), Scales (*Coccoidea*), and Medfly *(Ceratitis capitata)* egg decomposition in citrus fruits using (Red, Green and Blue) RGB couple-charged (CCD) cameras as well as other optical sensors through a system that detects several defects in citrus fruits. In the first study by Blasco et al. [40], acquired images were analyzed in the XYZ, H.S.I. (hue, saturation, intensity), International Commission on Illumination color standard-CIE L*a*b*, and L*u*v* color spaces in addition to RGB index, and only the color features were considered. The linear discriminant analysis (LDA) classifier was utilized and the classification rates for the defected fruits were in the range of 43.2–78.1%. In the second study by Blasco et al. [41], an unsupervised defect detection algorithm, called a region-oriented segmentation algorithm (ROSA), was developed to identify the symptoms of various diseases and defects in citrus fruits. This algorithm depends on creating different segmentation of the image of the fruit surface based on the color homogeneity of each region, and each segment grows by taking the neighboring pixels until healthy and defected areas are totally segmented. The referred algorithm enhanced the classification performance of defected fruits to 93.4–100%. Later, Blasco et al. [42] developed a system to identify 11 types of external citrus defects, among which are the Thrips, Scales, and Medfly egg decomposition. Along with a visible camera, there were two other cameras acquiring images in the NIR region and using a fluorescence light source. ROSA was also used to segment regions and then the Bayesian discriminant classifier was applied. The ratios of the defected fruits correctly classified were 73–86%. In another study carried out by López et al. [43] to detect the level of infestation on the citrus surface, an RGB camera with a fluorescence light source was used. Image processing was conducted using the multivariate image analysis algorithm developed by López et al. [44], which mainly depends on unfolding each image of the healthy samples to each R, G, and B channel, then intensity is transformed using a window of a certain size (3 × 3 or 5 × 5, etc.). Principle component analysis (PCA) was first implemented on the RGB images for feature extraction. T^2^ value, which implies the Mahalanobis distance of pixel neighborhood, was computed for each pixel in the set of training images and a value of T^2^ threshold was set. The process was applied for test samples’ images and the T^2^ was compared with the T^2^ threshold and a T^2^ map was created. The Bayesian discriminant classifier was then applied and yielded a classification rate for defected samples up to 92.8%.

Although the listed studies (Table 1) mainly focused on citrus fruits and two types of insect infestation, it is also clear that the idea of using color images to detect surface defects is effective as long as the infested tissue has different color or texture properties. Nonetheless, the use of visible color vision is not beneficial for the detection of internal defects as such problems cannot be recognized [47,48]. Moreover, some symptoms of surface infestation cannot be accurately detected with a color vision camera because of interference from the sample’s surface color. This requires using a more accurate and wavelength-based technique, such as hyperspectral or multispectral imaging systems [47,48].

### 3.3. Imaging Techniques

#### 3.3.1. Hyperspectral Imaging Systems

The hyperspectral imaging (HSI) technique is a relatively recent approach that is gaining extensive use in the agricultural production systems and food processing for noninvasive detection of properties and classification into quality categories. In the past decade and a half, it is among the most widely studied techniques for noninvasive monitoring of quality and ensuring the safety of fruits, vegetables, and food products [3,10,49,50,51,52,53]. The result of a sample scanning using the HSI system is a data cube (hypercube), where two (x and y) dimensions represent the spatial coordinates and the third dimension (λ) represents the wavelength coordinate [3]. The spectral responses can be related to the physical and chemical constituents of different agricultural products.

Detecting insect infestation in fruits and vegetables is an important application of this technique because it increases the accuracy of detection beyond random sampling, which is the common practice currently, reduces the risk of shipping infested samples, and decreases the cost of processing expended on an undetected infested sample that enters the supply chain. The HSI technique has the capability of accurate detection of hidden internal damage from insect infestations without sample destruction.

**Figure 1:**
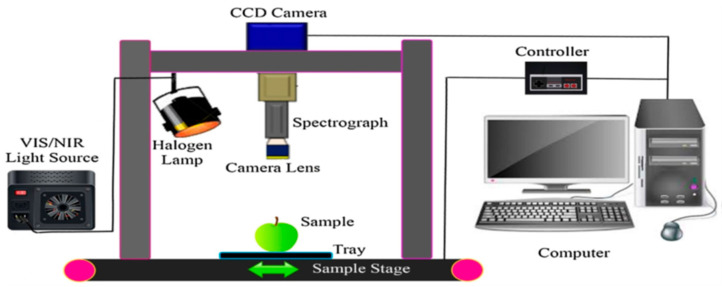
The components of a push-broom hyperspectral imaging system [10].

**Figure 2:**
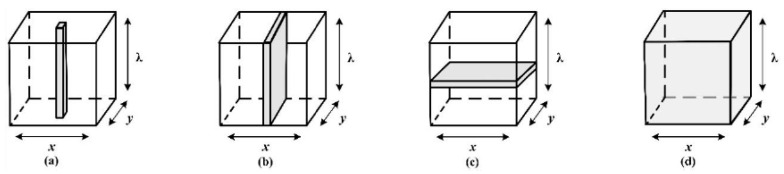
The basic hyperspectral imaging scanning modes: (**a**) point scanning, (**b**) line scanning, (**c**) area scanning, (**d**) single shot. x and y represent the spatial coordinates, λ represents the wavelength [3].

The main components of an HSI system are a light source in the visible and NIR ranges, a wavelength dispersive device, which is also called a spectrograph, and a camera that is either a charge-coupled device (CCD) or a complementary metal-oxide semiconductor (CMOS). Data acquisition occurs in different scanning modes. Figure 1 shows a complete set-up example of the push-broom HSI system [10]. The most common mode of acquiring data via an HSI system is the line scanning or push-broom mode (Figure 2b) [3]. The other three modes of HSI scanning, point scanning, area scanning, and single shot, are shown in Figure 2a,c,d.

**Figure 3:**
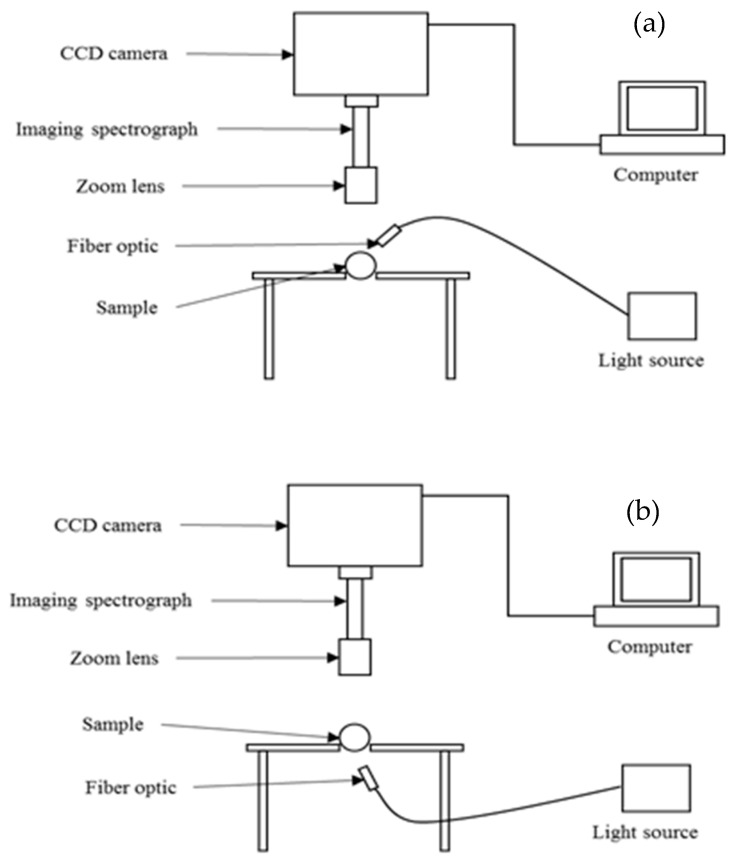
Schematic diagrams of hyperspectral imaging set-up in: (**a**) reflectance mode and (**b**) transmittance mode [31].

In a push-broom/line scanning system, the imaging system conducts a line-by-line scanning (could be by pixel or by depth) from the entire field of view and generates a two-dimensional image at the end of each scanning, such that the first dimension contains spatial information and the other one provides a full spectrum from a specific spot on a sample. Also, spectra can be collected in different forms, namely reflectance, absorbance/transmittance, and interactance modes. Each form is chosen based on the type and dimension of the sample and the position of the light source, spectrograph, and the camera [31,54,55]. Figure 3 shows a schematic diagram of the positioning of these components in the transmittance and reflectance HSI technique.

In order to normalize reflectance spectra to obtain relative reflectance (Equation (1)), a standard white reference is used to represent maximum reflectance, and by blocking the light source or scanning a complete dark surface, the minimum reflectance is obtained:

(1) $$R_{\lambda }=\frac{M_{\lambda }-C_{\lambda }^{0}}{C_{\lambda }^{1}-C_{\lambda }^{0}}$$

where *R_λ_* is the normalized/relative reflectance (%), *C*^0^ is the background (dark) intensity (counts), *C*^1^ is the reference (white) measurement intensity (counts), M is the sample’s measured reflectance intensity, and *λ* is a specific wavelength (nm). By normalizing the imaging spectral data, all sample spectral measurements are placed somewhere between the minimum and maximum intensity [49,56]. This normalizes the error that may ensue as a result of the change in intensity of illumination source during scanning.

In order to reduce the dimensionality of hypercube data for a quicker analysis and feedback process, and also to increase the potential application in online/inline settings, certain mathematical approaches, like partial least square (PLS) [31], stepwise discrimination analysis (SDA) [57], genetic algorithm (GA) [31], Bayesian discriminant analysis [15], sequential forward selection (SFS) and sequential backward selection (SBS) [10], and soft independent modeling of class analogy (SIMCA) [58], are applied for feature selection. Determining those wavelength regions allows for building a much simpler model, called a multispectral model. Multispectral imaging systems use the same principle of operation as the HSI systems, with the difference being fewer wavelengths, which accelerates data analysis and decision processes.

The application of HSI systems for detecting fruits and vegetables infested with insects has shown some promising results, even though there are more variations of targeted insects. Table 2 shows some of the recent studies where the HSI system was used for detecting insect infestation in fruits and vegetables. Several studies have researched insect infestation of citrus fruits using visible/near-infrared (Vis/NIR) HSI systems. A study conducted by Li et al. [59] applied an HSI system (400–1000 nm) to detect insect damage in citrus fruits. Principal components analysis (PCA) was used for dimension reduction and the band ratio algorithm was then used for classification. The classification rate was 100% for scale-infested samples. 

In other studies, the detection of insect infestations in mango fruits was also investigated. Saranwong et al. [15] studied the application of HSI (400–1000 nm) to assess fruit fly larvae infestation in mango. Reflectance spectra obtained were fed into a discriminant analysis classifier and the classification rate for infested and healthy fruits was up to 99.1% and 94.3%, respectively. It was found that the longer the post-infestation time, the easier the detection and the higher the classification rate is, which was attributed to the more visibility and intensity of symptoms of infestation with time. Haff et al. [62] also studied the same insect in mango using the same system. These researchers developed an algorithm to identify and mark the infested areas using four steps: background removal, Gaussian blur, thresholding, and particle counting. Discriminant analysis was applied, and the classification rates reached 99% for infested samples.

The identification of external insect infestation of jujube fruits was investigated by Wang et al. [57] using the visible range of HSI (400–720 nm). The relative reflectance spectra were extracted for each image and a stepwise discriminant classifier was applied. The classification rates for infested and healthy fruits were 98% and 94%, respectively. On the other hand, Liu et al. [61] utilized the NIR region of HSI (900–1700) to detect a fruit moth (*Carposina niponensis* walsingham) infestation in Jujube fruits. Relative reflectance was also determined for each image and the band ratio (BR) algorithm was applied for classification. The rates of classification for healthy and infested fruits were up to 100% and 93.1%, respectively. The most influential wavelengths were found to be 987, 1028, 1160, 1285, and 1464 nm. Huang et al. [54] used the Vis/NIR HSI (400–1000 nm) in the transmittance mode to detect insect infestation on vegetable soybean (green soybean seed). They applied the support vector data descriptor (SVDD) on the relative transmittance spectra and determined classification rates for healthy and infested samples to be 97.3% and 87.5%, respectively. The work on vegetable soybean was expanded by Ma et al. [64] to include automatic selection of the region of interest (ROI) based on threshold segmentation. They performed wavelengths selection using a fuzzy-rough set model. The SVDD classifier was applied and the classification rates boosted to 100% and 91.7% for healthy and infested samples, respectively. The optimal wavelengths were found to be 705 and 943 nm using entropy characteristics, and 692, 743, and 975 nm for both energy and mean characteristics. Rady et al. [10] applied push-broom reflectance Vis/NIR HSI (400–1000 nm) to detect codling moth larvae in GoldRush apples. They applied several classifiers on the relative reflectance including LDA, partial least square discriminant analysis (PLS-DA), feed forward artificial neural networks (FFNN), decision tree (DT), and K-nearest neighbors (Knn). The highest classification rates were 81% and 86% for healthy and infested fruits respectively, using the DT classifier. Wavelength selection was performed using the sequential forward selection technique that led to the following wavelengths, 434.0, 437.5, 538.3, 582.8, and 914.5 nm to be selected for codling moth infestation detection and classification in GoldRush apples.

The studies profiled provide various levels of accuracy, demonstrating the potential application of HSI as a diagnostic, detection, and classification tool for various types of insects in fruits and vegetables in real-time systems. Because insect infestation happens deep inside the fruit or vegetable, it is challenging to recognize the issue using RGB-based machine vision. The HSI technique is more appropriate. The exploration of this technique is becoming more popular because of continuous price reduction in hardware to build a system, the increasing computing power of systems to handle big datasets, and the noninvasive usefulness in agricultural applications. HSI systems measure the light intensity at several wavelengths from visible to near infrared. Among these many wavelengths, a few of them that are useful are selected for building a model that can predict infestation. These wavelengths are usually figured out using machine learning statistical approaches such as PLS, SDA, GA, and so on. In spite of this promise, HSI technology is still not very rampant in commercial applications with regards to insect infestation detection. One of the limitations of HSI is the accuracy of detection or classification (Table 2). There are some applications where 100% accuracy is a must—for example, in fruits for the international market where failure can have a far-reaching effect. This challenge is being addressed with some new and more effective analytical approaches, like deep learning, bagging, and boosting, and the potential to further increase the accuracy of HSI measurement is waiting to be further explored [65,66,67,68]. Also, while the major steps (data acquisition, preprocessing, calibration, validation, dimensionality reduction, re-calibration, and re-validation) in developing an HSI solution are well defined, there is no simple way to determine the most effective mathematical-analytical approach needed for some of these. It is mostly trial and error to determine the most effective algorithm or model. There is a need to address this challenge going forward. The appropriate algorithm is determined case-by-case, even though several approaches, such as principal component analysis, used for size reduction, and LDA and artificial neural networks, are often implemented in classification tasks.

#### 3.3.2. X-ray Imaging

The principle of an X-ray imaging system is based on the transmission imaging technique in which an X-ray beam emitted from a source penetrates an object and attenuates based on the density variance of the object. The attenuated energy that passed through the object is detected using a photodetector, a film, or an ionization chamber on the other side. The attenuation coefficients of the object components lead to different contrast between such components [69,70,71]. Computed Tomography (CT) X-ray imaging is a more recent and advanced technique than plain X-ray technology. The latter technique solves the problem of having overlapping layers of soft tissues or complex bone structures [71]. The source and detector rotate around the object to generate an enormous number of 2-dimensional slices or images, which are used to create a 3-dimensional image called a tomogram [26,71].

X-ray imaging falls within the electromagnetic spectrum with a wavelength range of 0.01 to 10 nm, which corresponds to the frequencies range of 30 to 30,000 Petahertz [72]. It has energy from 0.12 to 12 keV with low penetration power, called soft X-ray, which has been explored as a non-destructive process for internal quality inspection of various agricultural products. Although the onset of the application of X-ray imaging was solely targeted to medical purposes—diagnostic and security inspection areas—using the system to detect defects and quality properties in agricultural commodities research commenced around the 1950s [73]. Because of the inherent limitations of X-ray (discussed in Section 6), its studies in agricultural products mainly focused on X-ray irradiation quarantine treatments [74] and on dry or lower water-containing materials, e.g., checking seed quality with soft X-ray radiography [75] and for detecting hidden infestation of crop plants. Because the grayscale of X-ray images is a function of the density and thickness of the test samples, the relative contrast of infestation spot to the intact region inside a typical fruit will vary. The gray intensity of X-ray images depends on the density and thickness of the test samples, so the relative contrast of the infestation site to the intact region inside a typical fruit varies with its position. In order to accurately determine whether a fruit has signs of insect infestation using an X-ray imaging analysis, an effective adaptive image segmentation algorithm based on the local pixels’ intensities and an unsupervised thresholding algorithm is developed.

**Figure 4:**
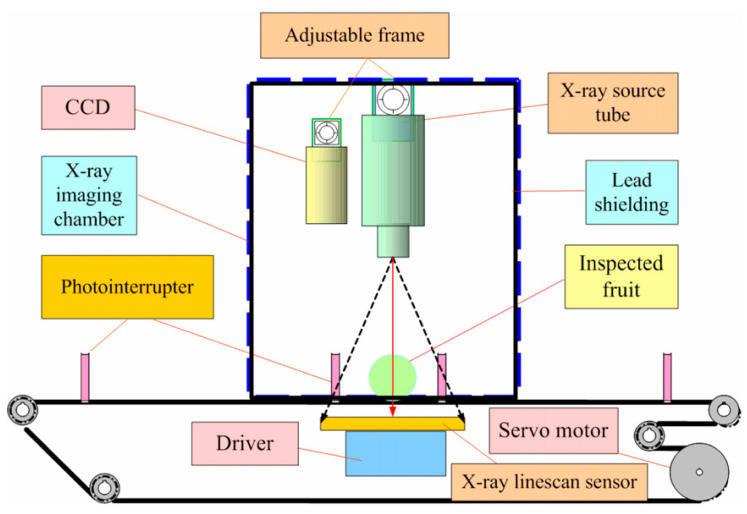
The layout of an X-ray quarantine scanning system [23].

The layout for architecture of an X-ray system developed by Chuang et al. [23] used for detecting insects in different agricultural produce is shown in Figure 4. The obtained values of 96.8%, 98.6%, 97.7%, and 98.7% were for sensitivity, specificity, accuracy, and precision, respectively. The mean time for scanning was 3.87 min per 100 fruits, which is an average of 2.3 s per fruit. Other studies where X-ray imaging was applied are profiled in Table 3.

**Figure 5:**
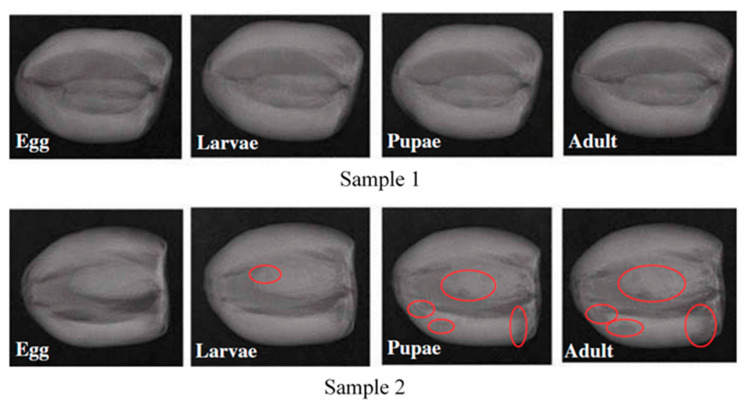
X-ray images of two samples of dates infested with *Oryzaephilus surinamensis* at different life stages. Samples 1 show infestation that is not visually recognizable, and samples 2 show identifiable infestation in areas circled in red [76].

Thomas et al. [85] used a medical-oriented X-ray radiography machine to assess the presence of seed weevil in mango. Images were evaluated visually, and no features were extracted. Later, Velasco and Medina [81] investigated the application of soft X-ray imaging to detect pulp weevil in freshly harvested green mango. Their results showed that the mango position affects the detection rates. Fruits scanned parallel to their shoulders showed less detection rates than those scanned perpendicular. Soft X-ray emission spectroscopy was also applied by Veena et al. [77] to detect fruit fly in Mango fruits. Color features were extracted from each image and differentiation between sound and infested fruits was feasible. However, no numerical values of the classification results were provided. Stone fruits—apples and cherries—were scanned for the response of codling moth and western cherry fruit fly respectively, using X-ray imaging by Schatzki et al. [82]. The tests performed included physical inspection of X-ray images by two operators on computers, and the results of both operators were averaged. Real-time sorting was stimulated by scrolling frames containing healthy and defected fruits on the computer screen at different rates, with the operators having the ability to identify the defected fruit on the screen. Classification rates for inspection tests were 0–96%, with the lower rates at the beginning of the infestation. However, such rates were much lower (8–58%) for scrolled frames. Haff and Pearson [79] utilized X-ray imaging to evaluate olive fruits for fruit fly. An algorithm for feature extraction was developed based on selecting 64 arbitrary features from each image. Iterative discriminant analysis was then used to select the optimal subset of only three features. Results showed that the best classification rates were 50–88%, with the lowest rates associated with fewer infestations. Haff et al. [78] developed a Bayesian classification algorithm to detect fruit fly on X-ray images of olive fruits. The same 64 features were used for Fruit fly detection in olives and discriminant analysis was applied to select the optimal set of three features. A 90% classification rate was obtained for healthy samples and 50–86% for infested samples. The feasibility of applying X-ray imaging for monitoring saw-toothed beetle in stored dates was studied by Al-Mezeini et al. [76]. The extracted features were 44 in total, based on the histogram and textural characteristics of the images (Figure 5). Linear discriminant analysis (LDA) was then performed along with bootstrapping for classification, and the best rates were 99% and 100% for healthy and infested samples, respectively. However, the infested tissue was not visually clearly differentiable from the healthy tissue, which tends to oppose the application of X-ray imaging in this case compared with other noninvasive systems such as HSI or spectroscopy that base their detection on differences in spectra formed by infested part and a healthy portion.

Another major downside of X-ray imaging is the large image dataset that impedes quick feedback time needed for the large quantity of produce often involved. This poses a challenge in online assessment and classification of insect-infested fruits and vegetables. Increasing computing power could significantly reduce the feedback time, then it adds significant cost to the technology. This is the improvement this technique needs.

#### 3.3.3. Magnetic Resonance Imaging (MRI)

MRI is a non-ionizing imaging technique in contrast to X-ray or computed tomography (CT) imaging and was first used for medical applications. The principle of MRI is such that a high-resolution image can be obtained by a strong and uniform magnetic field applied to hydrogen nuclei that are mainly located in water [71]. The image is formed as a result of the different levels of contrast of the object tissues as a response to a vigorous magnetic field and radio frequency waves. Applications of MRI in food quality monitoring is still considerably limited mainly due to the high cost of MRI systems. Torres [87] studied the application of a low-field MRI system to detect fruit fly in peaches, with classification rates of 58% and 71% for healthy and infested fruits, respectively. Haishi et al. [86] applied a low-field MRI using a 0.2 Teslaa or T magnet field to track the presence of peach fruit moth on apple fruits by analyzing multi-slice two-dimensional (2D) images. It was shown that the detection of larvae inside the fruit is feasible using a single slice gradient echo method in 6.4 s. Whereas, the multi-slice 2D measurement provided 6 images in 2 min, and these images covered a larger image area in a short time. Although MRI technology has a promising possibility for an effective noninvasive determination of fruits and vegetable defects, several problems still arise, especially when compared to other noninvasive systems such as color vision, hyperspectral and multispectral imaging, and spectroscopic systems. Such problems include the high cost for building, running, and maintenance, and the large volume and heavy weight of the MRI systems [86,90].

#### 3.3.4. Thermal Imaging

Thermal imaging (TI) is a sensing technique that was first illustrated for military applications. Later, TI was extended to agriculture and food process monitoring [71]. A typical TI system consists of a thermal camera that has an infrared detector, a signal processing unit, and an image acquisition unit [91]. The main idea of forming a TI image is based on the difference in surface temperatures radiated by an object that is linked to the thermal energy values. Such values are translated to electrical pulses which are processed in the signal processing unit to form an image. The same image segmentation approach applied in X-ray imaging to localize the infested region of interest is applied to thermal imaging.

Hansen et al. [88] used an infrared camera that was sensitive to 7.5–15 μm wavelengths to track codling moth in apple fruits. Data analysis was conducted via paired *t*-test and the results showed a significant difference (at α = 0.01) between healthy and infested fruits. In their thermal images, the infested area appeared to be slightly colder than the un-infested tissue. Detecting the infested area was not affected by the storage temperature nor the infestation location. Chen et al. [92] used an android powered TI system based on an Otsu image processing algorithm to detect maize tumor powdery mildew. Their goal was to use the segmentation result as a reference guide in unmanned area vehicles for precision spraying of pesticides. Chelladurai et al. [92] applied a thermal imaging system to delineate *Callosobruchus maculatus* (F.) (Cowpea seed beetle) infestation in mung bean and reported an accuracy of up to 80% detection using a machine learning approach.

Although TI is a promising non-destructive technique that can be effectively applied for insect detection in fruits and vegetables, either in the field or post- harvest, the sensitivity of a TI system is affected by the weather condition and the relatively high cost required to obtain a high-resolution thermal imaging camera. Combining the TI system with another noninvasive system such as color vision might enhance the sensitivity to weather conditions [93].

## 4. Acoustic Techniques for Insect Infestation Detection

Acoustics is the study of sound, which is generated by propagating mechanical waves of energy through an elastic medium by causing particle displacement and vibration. One of the most popular acoustic techniques used in agricultural product processing is ultrasound [94]. Insect pests usually bore deep into vegetable/fruit where other techniques may not be able to detect the infestation. Acoustic detection of insect activities is based on distinct sounds made by the larvae displacement when they are feeding or biochemical reactions in the pest-infested food that creates low-intensity ultrasonic sounds [17,95]. For example, crawling and feeding of two insects *Callosobruchus chinensis* and *Callosobruchus maculatus* in chickpea (*Cicer arietinum*) and mung bean or green gram (*Vigna radiata*) were monitored using a condenser-type microphone probe, with a frequency range of 20–16 KHz, placed inside an acoustic-proof bin [96]. Their results provided sound signatures for *Callosobruchus chinensis* and *Callosobruchus maculatus* insects as having a sound duration of 59 and 68 ms, and amplitude of 79.32 and 97.65 dB in chickpea and 84.01 and 95.53 dB in green gram, respectively. Moreover, they selected formants, formant bandwidth, frequency, and spectral power as principal features in their analysis for the infestation detection. They concluded that their method can be used for non-destructive early detection of insect infestation in bulk stored foods.

**Figure 6:**
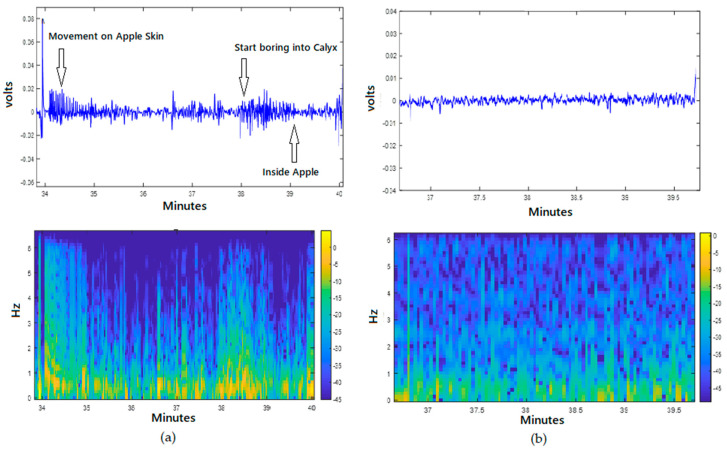
Acoustic signals from (**a**) infested versus (**b**) non-infested Apple samples [105].

Acoustic emission (AE) is one of the recently evolved areas of acoustics that can detect and monitor hidden insects and their activities in plants [97]. AE is the phenomenon where acoustic (elastic) waves are generated and radiated in solids that occurs when a material undergoes irreversible changes in its internal structure [98]. Differing from the conventional signaling techniques, AE can detect the physical signals produced from food crops to foodborne bacteria. Yang et al. [99] established the relationship between AE and crop disease stress, which allowed the detection of diseased crops from healthy ones. A highly sensitive AE device is capable of detecting the signal emitted by *Escherichia coli* and *Lactococcus lactis*, ssp. during their growth phases [97,100]. Ghosh et al. [101] used an AE system to acquire real-time data on *L. lactis*, ssp. metabolic activity and to dynamically monitor phase infection of cells. Application of AE for vegetable and fruit quality and safety assessment, specifically for insect activity detection, has been limited. Some previous studies showed that acoustic devices could be optimized to predict watermelon firmness [102], and to classify extruded bread with different water activity [103], and a contact AE detector was applied to evaluate apple texture with mechanical destruction of apples [104]. However, most of the studies that applied an AE technique are associated with food quality attributes by mechanically destroying the food. In a recent study, Li et al. [17] reported that AE detected codling moth activities in infested apples and they obtained a very high classification rate (83%) utilizing 0.5 s of acoustic signal collection. Also, an attempt was made by Ekramirad et al. [105] to authenticate the specific source and signature of acoustic emission in codling moth-infested apples through correlating visually observed larvae activities, such as chewing and locomotion, with patterns in the synchronized signals from the contact Lead zirconate titanate (PZT) sensors. For this objective, they video recorded the larval behavior while the acoustic signals were being collected at the same time. They found that larvae activities resulted in transient and quasiperiodic signals with frequency content lower than 6 Hz, where chewing signals show a rate of 1 to 2.3 times a second, while internal movement signals showed a large transient spike at irregular intervals. In Figure 6, it is shown that the signals from infested samples (on the right) are clearly different from the ones from non-infested samples. These results, they said, are not conclusive but they can authoritatively claim that there is a strong correlation between vibro-acoustic signal patterns and activities of the larvae within the apple fruits.

**Figure 7:**
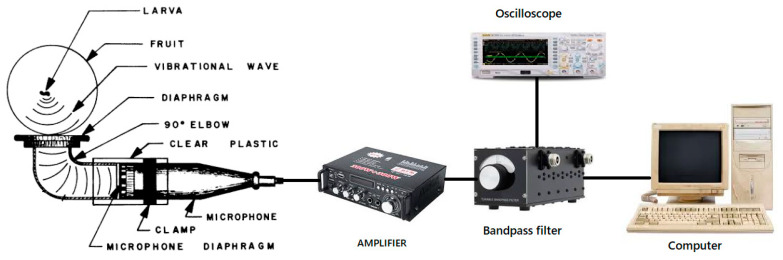
A typical acoustic emission system (Adapted from Litzkow et al. [109]).

The use of acoustic technology to replace labor-intensive and less-effective detection and monitoring methods on insect activity started to expand in the last three decades [19]. Acoustic technology has successfully detected the presence or absence of target insects [106], estimated the population density [107], and mapped insect populations [108]. The original AE system for detecting fruit fly (*Drosophila*) larvae activity was depicted by Litzkow et al. [109], which is shown in Figure 7. Nowadays, an AE system is usually composed of acoustic sensors, preamplifier, an input-output (I/O) board, and signal preprocessing software [17]. The acoustic sensor (diaphragm) serves as the device for collecting sound signals (Figure 7), and it is placed in direct close contact with the surface of the sample, thus the acoustic signal is propagated from the sample to the sensor [110]. The sensor sensitivity can vary from 40 to 100 kHz [109,111]. A reference sensor can be used to identify background or electrical noise since the sound generated by an insect is of higher energy [19].

**Figure 8:**
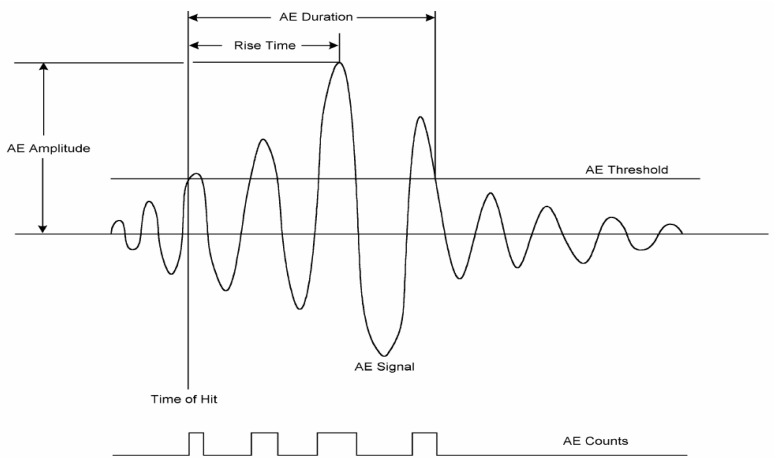
A typical acoustic emission signal diagram.

A signal triggered by the sensor is amplified through the preamplifier and digitized by the I/O board that serves as an oscilloscope. Signal amplification can range from 40 to 100 dB to minimize noise depending on the signal strength from larvae. Amplifiers can also eliminate extremely low- and high-frequency noises with appropriate filters [109]. Much of the background noise can be discarded by high-pass filters to remove long-duration and low-frequency background noises [112]. Filters can also help differentiate insect larvae at different development stages. Jalinas et al. [113] found that younger larvae have a shorter duration cycle than older (>30 days) larvae. Movements of insects are expected to generate impulses with a broader, higher-frequency spectrum than low-energy movements [114]. Signals acquired after amplification have a good signal-to-noise ratio [19]. The digital signal is then processed by commercial signal processing software to extract AE features. Common AE features derived from the original signal in previous literature include events and mean amplitude [115], duration, peak amplitude, rise time, ring down count, and event gap [116], energy rate [101], and larvae burst rate [113]. These are time-domain (also called temporal) AE features. A typical time-domain AE features’ derivation is shown in Figure 8. Through Fast Fourier Transform (FFT), frequency domain (also called spectral) features such as frequency range [117], frequency centroid, and peak frequency can be obtained [17].

For example, the frequency band most sensitive for insect detection was found to be from 0.4 to 1 kHz [118] through the analysis of signal spectrogram. The more features are extracted, the higher the probability to detect insect infestation from fruits and vegetables. In the study by Li et al. [17], 11 features (rise time, counts, energy, duration, amplitude, peak frequency, frequency centroid, average signal level, root mean square of signal, signal strength, and absolute energy) were collected from codling moth-infested apples. However, a higher quantity of features requires more computation power during signal processing and data analysis. Moreover, not all the features contribute to the detection of insect infestation, and this reduces the concern of higher computational power. In their study, Li et al. [17] determined that only 8 out of the 11 features are important in insect infestation detection.

For data analysis, most researchers applied simple statistical models (analysis of variance (ANOVA), multiple mean comparison) based on acquired AE features [101,102]. Powerful data analysis methods have been shown to enhance the detection performance of insects. Some advanced statistical and mathematical methods have been used and achieved success in recent years. Pinhas et al. [119] used Gaussian mixture modeling to obtain a detection ratio as high as 98.9%. Trifa et al. [120] used hidden Markov models to realize a species recognition rate of 99.5%. Li et al. [17] applied adaptive boosting to achieve a classification rate of 100%. Novel models for data analysis from insect AE data can be adapted from novel human speech recognition models, such as recurrent and convolutional neural networks [121]. It is expected that these advanced and novel models can improve insect acoustic recognition performance.

The promise that AE portend for insect infestation detection is strong, with a high detection rate that needs marginal improvement for industrial deployment, and a short signal collection time for a quick system feedback. However, it is not still clear the exact source of sound being detected that allows for differentiation between healthy and infested fruits. The literature has conflicting reports on the source of sound [101,109,122]. Future studies should address the speculation that this is due to insect displacement, or, it could be due to biochemical reactions where gas implosion is creating the peculiar sounds being detected.

The relatively recent Laser Doppler Vibrometer (LDV) technology was developed for measuring the surface vibrations in a non-contact manner. The LVD sensor emits a laser beam towards the surface of the vibrating specimen in order to extract the vibration amplitude and frequency from the Doppler shift induced in the incident laser beam [123]. One of the first attempts to apply the LDV method for the detection of insect infestations, based on larval feeding and locomotion vibrations in the specimen, was conducted by Zorovic and Cokl [124]. They used a portable digital laser vibrometer (PDV-100, Polytec GmbH, Waldbronn, Germany) to detect the activity of Asian long-horned beetle (*Anoplophora glabripennis*) in logs of Populus. The log samples were prepared by fitting a 4 mm^2^ reflective tape in the point of laser beam incident in order to receive a good reflection of the laser beam. Their results revealed that there were three types of vibrational pulses: low-frequency pulses, high-frequency pulses, and broadband pulses. They hypothesized that the broadband signal was correlated to larvae activity of biting the wood fiber, while the low-frequency and high-frequency pulses were related to non-feeding movements. Their results confirmed the ability of the LDV method to detect insects in wood, but they did not provide the exact size, position, or number of insects inside the specimen. In another study, the LDV system (Polytech PDV-100 Digital Laser Vibrometer) was used to direct the laser beam on the surface of rice within the test container in order to detect the *Trogoderma inclusum* and *Tenebrio molitor* infestation [118]. They concluded that the system was not very promising in the detection of small insects in grains, due to the need for getting the insect as close as two inches to the laser beam incident point. As a non-contact method, this technique is advantageous in terms of avoiding the interference between sensor and sample, not being affected by background noises, no need to attach the sensor on specimen, and covering a wide frequency range and working distance [8,124]. On the other hand, the application of this method is currently limited because of poor results for the specimens with rough surface, which is not a good reflector of laser beams as well as being susceptible to the so called ‘speckle noise’ [125]. Thus, further studies are needed to expand the application of this method in a variety of agricultural products.

## 5. E-Nose and E-Tongue

Early-stage detection of insect infestation on vegetable/fruit production and logistics is highly desirable to reduce economic loss and to ensure food safety. When vegetable and fruit are under insect attack, the physical, chemical, and biological changes are difficult to determine. Electronic nose (E-nose) and tongue (E-tongue) technologies are effective in determining these changes by applying biosensors to qualify and quantify the changes [126]. E-nose works as artificial olfaction devices that mimic the mammalian olfactory system. Both devices are composed of non-selective or semi-selective sensors interacting with aromatic or tasty compounds to produce electronic signals [126,127]. E-noses have been successfully used for the detection of insect-infested fruits and vegetables, and insect population dynamics [128]. Under different growth conditions, tomato plants infested with spider mites (*Tetranychus urticae* Koch) were correctly classified without a prior knowledge based on the volatile organic compounds (VOCs) profiles emitted by infested tomato plants [129]. E-nose has demonstrated the ability to precisely predict the gender and species of stink bugs [130,131].

**Figure 9:**
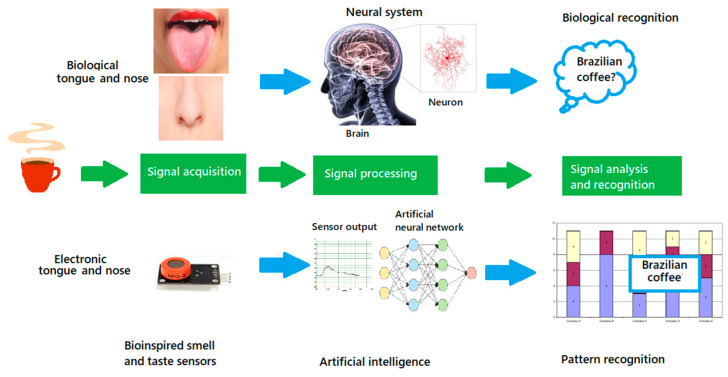
A schematic diagram illustrating E-nose and E-tongue in comparison with biological nose and tongue (Adopted from Wang et al. [132]).

A schematic diagram illustrating E-nose and E-tongue in comparison with biological noses and tongue is shown in Figure 9. An E-nose or E-tongue system is composed of two main components: a sensing system and a signal processing system [132]. The most commonly used sensors in E-nose include metal oxide semiconductor (MOS) sensors, conducting polymer (CP) sensors, optical sensors, and piezoelectric sensors [132]. The most commonly used sensors in E-tongue include electrochemical (potentiometric, voltammetric, amperometric, impedimetric, and conductimetric), optical, mass, and enzymatic sensors (biosensors) [49,132]. Fundamentals of different types of sensors for E-nose and E-tongue are explicitly explained by Wang et al. [132]. In the process of detection, reversible physicochemical changes to the sensing materials are triggered and electrical properties such as resistance and electrical potential will change [128]. Sensors measuring the resistance and electrical potential include conductivity and gravimetric sensors. The conductivity sensor is based on a conducting polymer and/or metal oxide semiconductor, both of which work on the principle of variations in conductivity or resistance. Gravimetric sensors are based on the wave produced along with or through the surface of the sensor. The working principle involves a change in the mass of the piezoelectric sensor coating which results in a change in the resonant frequency [133]. The operation of an optical sensor is based on the changes in chemical properties, such as reactivity, redox potential, and acid–base interactions [134]. Optical sensors use a wavelength-selectable light source, a light detector, and sensor materials that interact with gases. Colorimetry and fluorometry are the two typical techniques used for analyzing the signal obtained from optical sensors [134].

One of the most applicable E-noses is based on the phytohormones and VOCs emitted by insects or insect-infested vegetables/fruits. Phytohormones and VOCs are defensive chemical messengers and substances respectively, which will change dramatically when fruits/vegetables are under attack [135]. Differential sensor arrays can transform the VOCs information into electrical signals. Similarly, insect antenna-based E-nose can also be a valuable tool for the detection of pest infestation [132]. One available commercial E-nose (PEN2) comprising 10 metal-oxide semiconductor (MOS) sensors successfully fingerprinted the VOCs present in insect-damaged samples [136]. The E-nose systems are manufactured by Win Muster Airsense (WMA) Analytics Inc. of Schwerin Germany [136]. Despite using commercial E-nose instruments, recently there have been some studies trying to self-design E-nose systems to match case-specific parameters of control and to avoid the costs of using general commercial devices. As an example, Wen et al. [137] developed a sweeping electronic nose system (SENS), composed of 8 metal-oxide-gas (MOS)-type chemical sensors, combined with PCA and LDA data processing methods to detect the early damage of oriental fruit fly (*Bactrocera dorsalis*) in mandarin (*Citrus reticulate Blanco*) citrus fruit. Their results showed that the SENS could classify the *B. dorsalis*-infested citrus fruits with a recognition accuracy of 98.2%. They concluded that more study is needed to analyze the effects of other pest invasion on VOCs emitted from citrus fruits, and to collect enriched data covering different levels of citrus fruit infested by *B. dorsalis*, different varieties, and ripe stages of citrus fruit.

The aforementioned studies used E-nose and E-tongue with promising results. The development of intelligent E-nose systems for the specific purpose of detection of insect in fruits and vegetables is the research direction of the future. Specific applications include the discrimination of insect species and gender, insect development stage, insect population dynamics, and damage status of fruits and vegetables. Biosensors are the key to the success of E-nose and E-tongue. Insect odorant receptor based on the sensor is sensitive at ppb levels [138]. However, fruit and vegetable processing involves high humidity, which shortens the sensor lifetime and deteriorates sensor performance [128]. Performance of the sensor is important for practical industrial applications, though there is a simple problem: it requires an enduring solution beyond using hydrophobic materials as substrates for the sensor manufacture, which in turn shortens its shelf-life.

## 6. Critical Comparison between Different Noninvasive Methods

Table 4 provides an overall comparison of non-invasive methods used for insect infestation detection in fruits and vegetables by summarizing their advantages and disadvantages.

## 7. Conclusions

The significance of ineffective insect infestation detection in fruits and vegetables is broad. It lies in the reduction in the value of produce that may ensue when they enter the supply chain without detection and control, the economic losses when infestation causes a ban of produce export, spread or damage occurring to high-quality produce, and the safety issues related to consuming or processing infested produce. This paper reviewed different methods that have been explored in the last few years for non-destructive detection and classification of fruits and vegetables infested with different types of insect pests. Agricultural production is at a scale and stage where subjective assessment is insufficient to meet the scale of quality needed by the industry. The development of highly sensitive and accurate technologies for performing the role typically done by human subjects is essential for quick turnover to meet regulatory and consumer demands. Several of the technologies available have prospects and limitations. Some of the challenges include the high cost of implementation, sensitivity, accuracy, feedback time, and in some cases, safety. Techniques such as hyperspectral imaging, electronic nose, and acoustic emission are emerging as the sensors needed for artificial intelligent system deployment to address this need. HSI especially has been applied as the baseline technology in some other industries, and its potential for success in insect infestation prediction is promising, so long as the accuracy is guaranteed. A lot of these techniques require a machine learning computational approach for development and deployment. Advanced machine learning approaches like sensor data fusion and ensemble machine learning have allowed for combining the strengths of different approaches, and models for better results have shown the potential benefits of improving the models for quality assessment of fruits, vegetables, and food products [147]. Current improvement in the analytical approach of big data and feedback speed will benefit these methods and make them more amenable.

## Figures and Tables

**Table 1:** Studies on detection of insect infestations in fruits and vegetables using visible color cameras.

Sensor Type	Crop	Insect Type	Machine Learning Technique	Classification Results	Reference
RGB camera	Citrus	Scale insect (*Coccoidea*)	MIA	92.8%	[43]
RGB camera	Citrus	Thrips (Thysanoptera), Scales, and Medfly (*Ceratitis* *capitata*) egg	BDA	73–86%	[42]
RGB camera	Citrus	Medfly	BDA	NA	[45]
RGB camera	Citrus	Thrips, Scales, and Medfly egg	ROSA	93.4–100%	[41]
RGB camera	Citrus	Thrips, Scales, and Medfly egg	LDA	43.2–78.1%	[40]
Line scan cameras	Pistachio	Insect damage	DF	74–91.8%	[46]

MIA: multivariate image analysis; BDA: Bayesian discriminant analysis; LDA: Linear discriminant analysis; ROSA: region-oriented segmentation algorithm; DF: discriminant function; RGB—Red, Green and Blue color spaces; NA—not applicable.

**Table 2:** Studies on insect infestations detection in fruits and vegetables using hyperspectral imaging systems (HSI).

Sensor Type: Wavelength, nm	Crop	Imaging Mode	Insect	Machine Learning Technique	Classification Results	Reference
HSI: 400–900	Apple	Reflectance	Codling moth	DT	Healthy: 81% Infested: 86%	[10]
HSI: 400–1000	Citrus: Orange	Reflectance	N/A	PCA and BR	100%	[59]
HSI: 450–930	Citrus: Red Ruby Grapefruit	Reflectance	Leafminers	SID	95.2%	[60]
HSI: 400–720	Jujube	Reflectance	External insect	SDA	Healthy: 98% Infested: 94%	[57]
HSI: 900–1700	Jujube	Reflectance	Carposina niponensis walsingham	BR	Healthy: 100% Infested: 93.1%	[61]
HSI: 400–1000	Mango	Reflectance	Fruit fly	DA	Up to 99%	[62]
HSI: 400–1000	Mango	Absorbance	Fruit fly	DA	Up to 99.1 %	[15]
HSI: 1000–1600	Mung bean	Reflectance	*Callosobru-chus maculatus*	LDA and QDA	Healthy: 93.7% Infested: 75.5–95.7%	[55]
HSI: 740–1000	Pickling cucumbers	Transmittance and Reflectance	Fruit fly	PLS-DA	88–93%	[21]
HSI: 580–980 and 590–1550	Tart cherry	Transmittance and Reflectance	Plum curculio	GA and PLS-DA	Healthy: 81.3% Infested: 95.8%	[31]
HSI: 460–800	Tomatoes	Reflectance	Tomato hornworms frass	Detecting algorithm	Healthy: 86–95% Infested: 71–99%	[63]
HSI: 400–1100	Tomatoes	Transmittance	*Tuta absoluta* (Meyrick)	ANN	95% Classification accuracy	[58]
HSI: 400–1000	Vegetable soybean	Transmittance	Etiella zinckenella Treitschke (moth)	SVDD	Healthy: 97.3% Infested: 87.5%	[54]
HSI: 400–1000	Vegetable soybean	Transmittance	Pod borer *(Maruca vitrata*)	SVM	Healthy: 100% Infested: 91.7%	[64]

ANN: Artificial Neural Network; BR: Band Ratio; DT: Decision Tree; DA: Discriminant Analysis; LDA: Linear Discriminant Analysis; QDA: Quadratic Discriminant Analysis; PCA: Principal Component Analysis; PLS-DA: Partial Least Square Discriminant Analysis; SID: Spectral Information Divergence; SVDD: Support Vector Data Description; SVM: Support Vector Machine; N/A: Not Available.

**Table 3:** Studies of detecting insect infestations in fruits and vegetables using X-ray imaging, MRI, and thermal systems.

Sensor Type	Crop	Insect	Machine Learning Technique	Classification Results	Reference
X-ray machine	Dates	Saw-Toothed Beetles (*Oryzaephilus surinamensis*)	LDA and SDA	Healthy: 99% Infested: 100%	[76]
Soft X-ray machine in IICPT	Mango	Fruit fly	N/A	N/A	[77]
X-ray cabinet	Olives	Fruit fly	DA	Healthy: 90% Infested: 50–86%	[78]
X-ray images	Olives	Olive fly(*Bactrocera oleae*)	IDA	50–88%	[79]
X-ray CT imaging and film X-ray	Apples Cherries	Codling moth and western cherry fruit fly (*Rhagoletis indifferens*)	N/A	N/A	[80]
Soft X-ray machine	Mango	Mango pulp weevil(*Sternochetus frigidus*)	N/A	N/A	[81]
Film and on-line scanning X-ray equipment	Apples	Codling moth	None—visual	6–99%	[82]
X-ray machine	Pistachio	Navel orange worm(*Amyelois transitella*)	SDA	40–67%	[83]
X-ray machine	Pistachio	Insect damage	ANN	54–96%	[84]
X-ray machine	Mango	Seed weevil (*Sternochetus mangiferae*)	N/A	100%	[85]
Low-field MRI equipment	Apples	Peach fruit moth (*Carposina Sasakii Matsumura*)	N/A	100%	[86]
Low-field MRI equipment	Peaches	Fruit fly	N/A	Healthy: 58% Infested: 71%	[87]
IR thermal camera	Apples	Codling moth	Paired *t*-test	Significant at α = 1%	[88]
IR Thermal camera	Cowpea	Cowpea seed beetle (*Callosobruchus Maculatus*)	QDA	80%	[89]

ANN: Artificial Neural Network; DA: Discriminant Analysis; LDA: Linear Discriminant Analysis; IR: Infrared; QDA: Quadratic Discriminant Analysis; MRI: Magnetic Resonance Imaging; SDA: Stepwise Discriminant Analysis; IDA: Iterative Discriminant Analysis; CT: Computed Tomography; N/A: Not Available.

**Table 4:** Merits and demerits of the different nondestructive methods.

Method	Advantages	Disadvantages
Spectroscopy	No sample preparation needed, determining both chemical and physical characteristics, ease of use and suitable for on-line applications [16,139]	Large amount of samples/data and different chemometric methods are needed to build accurate models [140]. Does not provide spatial data.
Visible light sensing	Simple and cost-effective, accurate and suitable for on-line monitoring [61,141]	Only suitable for detecting external defects, sensitive to external lighting variations
HSI	Merges the advantage of a color vision system with that of spectroscopic system [142]. Provides both spectral and spatial features for accurate segmentation and identification of region of interest, it can detect internal defects [140]	HSI data are voluminous, contain huge redundant data that requires tedious analysis to upgrade to multispectral images by selecting useful wavelengths), its hardware is costly, different chemometric methods are required to extract useful information [140]
X-ray imaging	Can detect internal defects causing density differences, such as cavities	High costs, poor penetration in materials with high water content, and difficulty in effectively differentiating normal and infested tissues with similar densities [143]
MRI	No harmful ionizing radiation, high-resolution visual information of internal structure, it gives quality 2D and 3D images [144]	High costs, large dimensions, and heaviness [86,90]
Thermal imaging	Easy handling and portability [144]	Sensitivity to the environmental condition and relatively high costs to obtain high-resolution images [93]
Acoustic	Sensitive, efficient, and clear detection capabilities of various insects [145]. Inexpensive, automatic, and continuous monitoring [19]	Prone to background noise [19]. Incapable of detecting insect eggs
E- nose and E-tongue	Low-cost, rapid, and environmentally friendly testing [146]	Reported detection levels and accuracies are not very high [146]
